# Corps étranger intra irien

**DOI:** 10.11604/pamj.2018.30.181.11218

**Published:** 2018-06-27

**Authors:** Saloua Azennoud, Mohamed Zarrouki

**Affiliations:** 1 Université Mohammed V Souissi, Service d’Ophtalmologie B de l’Hôpital des Spécialités, Centre Hospitalier Universitaire, Rabat, Maroc

**Keywords:** Corps étranger, iris, yeux, Foreign body, iris, eyes

## Abstract

We report the case of a 28-year old man victim of left ocular trauma. Ophthalmologic examination showed visual acuity 8/10 (without correction), cloudy patch at 7 o’clock (A) with foreign body in the iris at the level of cloudy patch (B).

## Image en médecine

Nous rapportons un cas d’un jeune homme âgé de 28 ans victime d’un traumatisme oculaire OG, l’examen ophtalmologique objectif au niveau de l’œil atteint une acuité visuelle à 8/10 sans correction, une taie cornéenne à 7h (A) avec un corps étranger intra irien en regard de la taie (B).

**Figure 1:**
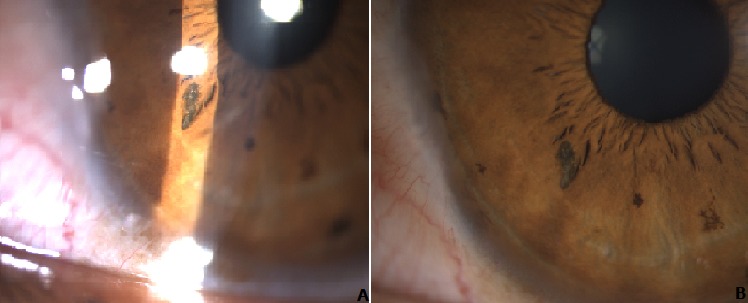
A) segment antérieur montrant une taie cornéenne à 7h de l’OG; B) segment antérieur montrant le corps étranger intra irien en coupe et au fort grossissement

